# Automated workflow-based exploitation of pathway databases provides new insights into genetic associations of metabolite profiles

**DOI:** 10.1186/1471-2164-14-865

**Published:** 2013-12-09

**Authors:** Harish Dharuri, Peter Henneman, Ayse Demirkan, Jan Bert van Klinken, Dennis Owen Mook-Kanamori, Rui Wang-Sattler, Christian Gieger, Jerzy Adamski, Kristina Hettne, Marco Roos, Karsten Suhre, Cornelia M Van Duijn, Ko Willems van Dijk, Peter AC 't Hoen

**Affiliations:** 1 Center for Human and Clinical Genetics, Leiden University Medical Center, S4-P, PO Box 9600, 2300, RC Leiden, Netherlands; 2 Department of Clinical Genetics, DNA Diagnostics Laboratary, University of Amsterdam, Amsterdam, Netherlands; 3 Genetic Epidemiology Unit, Departments of Epidemiology and Clinical Genetics, Erasmus University Medical Center, Rotterdam, Netherlands; 4 Department of Physiology and Biophysics, Weill Cornell Medical College in Qatar, Education City, Qatar Foundation, PO Box 24144, Doha, State of Qatar; 5 Research Unit of Molecular Epidemiology, Helmholtz Zentrum München, German Research Center for Environmental Health, Neuherberg, Germany; 6 Institute of Genetic Epidemiology, Helmholtz Zentrum München, German Research Center for Environmental Health, Neuherberg, Germany; 7 Institute of Experimental Genetics, Genome Analysis Center, Helmholtz Zentrum München, German Research Center for Environmental Health, Neuherberg, Germany; 8 Chair of Experimental Genetics, Technische Universität München, Munich, Germany; 9 Institute of Bioinformatics and Systems Biology, Helmholtz Zentrum Munchen, German Research Center for Environmental Health, Neuherberg, Germany; 10 Department of Endocrinology, Leiden University Medical Center, S4-P, PO Box 9600, 2300, RC Leiden, Netherlands

**Keywords:** Genome-wide association, Metabolite, Genotype-phenotype prioritization, Bioinformatics, Pathway databases

## Abstract

**Background:**

Genome-wide association studies (GWAS) have identified many common single nucleotide polymorphisms (SNPs) that associate with clinical phenotypes, but these SNPs usually explain just a small part of the heritability and have relatively modest effect sizes. In contrast, SNPs that associate with metabolite levels generally explain a higher percentage of the genetic variation and demonstrate larger effect sizes. Still, the discovery of SNPs associated with metabolite levels is challenging since testing all metabolites measured in typical metabolomics studies with all SNPs comes with a severe multiple testing penalty. We have developed an automated workflow approach that utilizes prior knowledge of biochemical pathways present in databases like KEGG and BioCyc to generate a smaller SNP set relevant to the metabolite. This paper explores the opportunities and challenges in the analysis of GWAS of metabolomic phenotypes and provides novel insights into the genetic basis of metabolic variation through the re-analysis of published GWAS datasets.

**Results:**

Re-analysis of the published GWAS dataset from Illig et al. (Nature Genetics, 2010) using a pathway-based workflow (http://www.myexperiment.org/packs/319.html), confirmed previously identified hits and identified a new locus of human metabolic individuality, associating Aldehyde dehydrogenase family1 L1 (*ALDH1L1*) with serine/glycine ratios in blood. Replication in an independent GWAS dataset of phospholipids (Demirkan et al., PLoS Genetics, 2012) identified two novel loci supported by additional literature evidence: *GPAM (*Glycerol-3 phosphate acyltransferase) and *CBS* (Cystathionine beta-synthase). In addition, the workflow approach provided novel insight into the affected pathways and relevance of some of these gene-metabolite pairs in disease development and progression.

**Conclusions:**

We demonstrate the utility of automated exploitation of background knowledge present in pathway databases for the analysis of GWAS datasets of metabolomic phenotypes. We report novel loci and potential biochemical mechanisms that contribute to our understanding of the genetic basis of metabolic variation and its relationship to disease development and progression.

## Background

GWAS have resulted in the identification of novel genetic loci associated with a variety of diseases and clinical phenotypes. However, a disease or clinical phenotype is the end point of the behaviour of numerous genes and pathways in addition to environmental influences. This at least partly explains the general observation that the effect size of genetic association with clinical phenotypes is rather small. Spurred by recent technological developments in the field of metabolomics, interest in genome wide association studies with metabolite levels in blood [1-4] is gathering momentum. Metabolites are intermediate phenotypes, entities that lie between genes and clinical end points [5,6]. Due to their proximity to an enzyme/gene, metabolites may offer greater effect sizes for GWAS than clinical phenotypes [7]. Moreover, the pathways in which the metabolite plays a role may provide insight into the underlying biological mechanism responsible for the development of the associated disease.

Typically, in metabolomics GWAS, hundreds of metabolites are tested for genetic association. However, association of all SNPs with all measured metabolites comes with considerable multiple testing problems. Recent publications have also shown that testing ratios of metabolites for genetic association results in much larger effect sizes; however this further exacerbates the multiple testing problem which precludes genuine SNP-metabolite pairs from reaching genome-wide significance. Several approaches like gene based tests [8,9] and pathway analysis [10] have been proposed to overcome this limitation of inadequate statistical power in GWAS. All these approaches have been suggested in the context of GWAS with clinical phenotypes but genetic association with metabolites presents its own set of unique opportunities and challenges. Herewith, we explore the utility of background knowledge present in metabolic pathway databases to increase the power in identification of metabolite Quantitative Trait Loci (mQTL).

Our approach involves selective testing of SNPs near genes in pathways supposedly relevant to the metabolite levels, as a way to reduce the multiple testing burden in GWAS. Background knowledge pertaining to a metabolite is retrieved through systematic interrogation of metabolic pathway databases which describe biochemical pathways, reactions, and enzymes relevant to human metabolism. Several pathway databases have been created by groups around the world, while the intent of these efforts remains the elucidation of biological mechanism, the databases however, differ quite significantly in their content, size, user accessibility, download formats and most importantly availability and type of web services for machine-enabled interrogation of the database [11]. In this publication, as a proof of principle, we have chosen to focus on two important metabolic pathway databases, KEGG [12] and BioCyc [13]. KEGG is an integrated database resource of seventeen databases which provide system, genomic and chemical information. The pathway database consists of both metabolic and non-metabolic pathways and is constructed by a team of curators based on information available in the literature. BioCyc is a collection of pathway/genome databases that describe the genome and metabolic pathways of several organisms. The database that describes human genomes and pathways, HumanCyc was interrogated in this study. In our approach, for every metabolite under consideration, genes acting in the vicinity of the metabolite are determined using knowledge present in databases mentioned above. We thus generate an integrated set of genes that represent entities with influence over the metabolite. A workflow management system called Taverna [14] was used to generate these gene sets and the SNPs associated with these genes. The workflows that were designed for this purpose have been submitted to a workflow repository at http://www.myexperiment.org/packs/319.html[15].

A previously published metabolomics dataset by Illig et al. 2010 [2] was analyzed to evaluate the sensitivity of the method in picking true positives and to identify novel SNP-metabolite pairs that had hitherto been obscured in the GWA list given the stringent threshold for significance. In addition to validating a novel bioinformatics workflow analysis tool, we identified a new locus of human metabolic individuality, Aldehyde dehydrogenase family1 L1 (*ALDH1L1*). This locus was found associated with serine/glycine ratios, a metabolic trait that functionally matches the gene function.

Candidate genes identified through the analysis of Illig et al. dataset were taken up for replication in a separate study published by Demirkan et al. [4]. We report *GPAM* (Glycerol-3 phosphate acyltransferase) and *CBS* (Cystathionine beta-synthase) as novel loci associated with phosphatidylcholine moieties.

## Results

**Figure 1:**
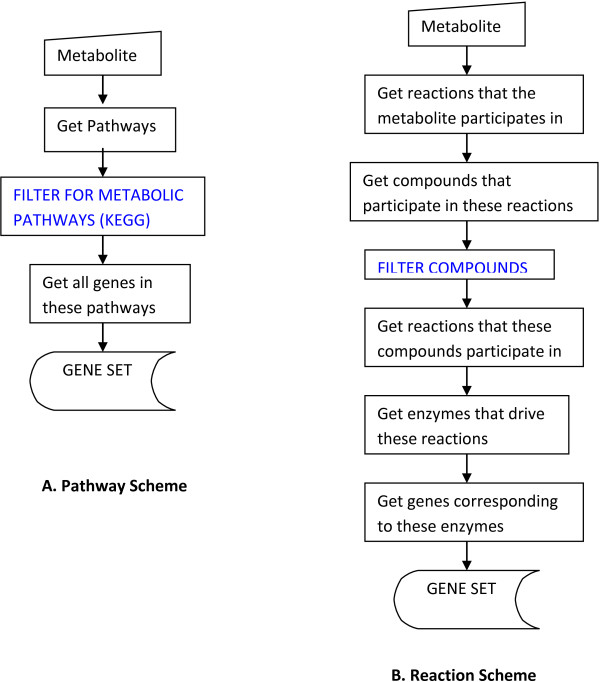
**The database interrogation schemes.** The two interrogation schemes: pathway scheme **(A)** and reaction scheme **(B)** are shown. The blue color indicates the intermediate steps to filter out certain pathways/compounds from the two schemes to avoid non-specific connections.

**Figure 2:**
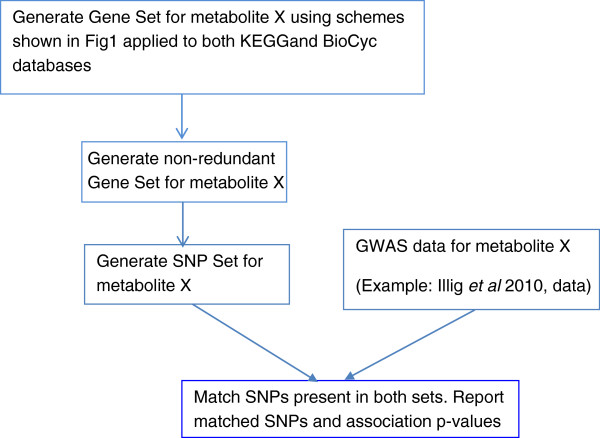
**Strategy to find biologically relevant SNP-metabolite pairs in published GWAS datasets.** Background knowledge pertaining to a metabolite is collected from the pathway databases KEGG and BioCyc in an automated fashion to generate a gene/SNP set relevant to the synthesis and degradation of the metabolite.

Our approach can be divided into three stages: (i) Generate a non-redundant gene set for every metabolite considered using knowledge in pathway databases like KEGG and BioCyc applying interrogation schemes as shown in Figure 1 and outlined below. (ii) For every gene in the set, generate the set of SNPs within the gene and 50 kb flanking sequences, and create a SNP set for each metabolite (iii) Match SNPs generated for a metabolite with the GWAS for the same metabolite and store the matches with the p-values reported for the association (Figure 2).

### Analysis strategy of databases and Interrogation schemes

To retrieve a prioritized list of candidate genes associated with metabolite levels, gene sets were generated for each metabolite through the pathway scheme and the reaction scheme [Figure 1A and 1B] for the KEGG and BioCyc databases (see Method). The pathway scheme generates a list of genes that participate in pathways relevant to the synthesis or degradation of the metabolite. In the reaction scheme, the metabolite is used as a seed node and shells of reactions around the metabolite are explored. The list of genes that catalyse the reactions are retrieved and form the gene set for the given metabolite. For every gene set, a corresponding SNP set is generated by retrieving SNPs within the flanking 50 kb of every gene. In the final step, the SNP set for a metabolite is matched with the GWAS dataset for the same metabolite. At this stage, the sensitivity of the method is evaluated and potential novel discoveries are explored.

**Figure 3:**
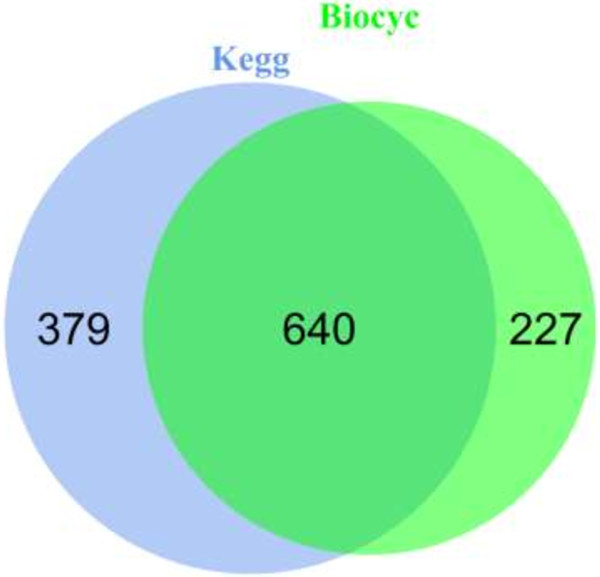
**Gene set overlap for the KEGG and BioCyc databases.** The Venn diagram depicts the overlap between the non-redundant gene set for KEGG and the BioCyc metabolic pathway database. These genes correspond to the combined set from the pathway and reaction interrogation schemes. The total number of unique genes that our method yields is **1246**.

Results for each of three classes of metabolites (14 amino acids, 1 carnitine and 2 lipids) are shown in Table 1. For example, for glycine, interrogation of the KEGG database identified 173 and 432 genes using the pathway and reaction schemes respectively, whereas the corresponding numbers of genes were 90 and 192 for the BioCyc database. The union of all the four interrogation schemes results in a gene set consisting of 523 genes relevant to glycine metabolism (Table 1). For all the three classes of metabolites, 1246 unique genes were found, 640 are common to KEGG and BioCyc, the number of genes unique to each of the two databases are 379 and 227 respectively (Figure 3).

**Table 1:** Gene and SNP sets generated by the database: interrogation schemes for each of the metabolites

**Metabolite**	**BioCyc Pathway**	**BioCyc Reaction**	**KEGG Pathway**	**KEGG Reaction**	**Size of unique gene set**^**1**^	**Size of unique SNP set**^**2**^	**Number of tests**^**3**^
Arginine	20	104	57	179	257	10788	10788
Glutamine	51	132	100	282	388	15591	15591
Glycine	90	192	173	432	523	20767	20767
Histidine	8	9	45	155	181	7126	7126
Leucine	8	0	44	83	117	5037	5037
Methionine	27	104	35	243	284	11532	11532
Ornithine	16	150	103	159	247	10089	10089
Phenylalanine	6	113	25	163	196	8419	8419
Proline	10	12	57	83	119	5075	5075
Serine	37	135	152	219	360	14996	14996
Threonine	1	11	39	49	75	2633	2633
Tryptophan	15	19	78	221	261	10419	10419
Tyrosine	14	106	61	158	219	9365	9365
Valine	15	93	80	137	211	9365	9365
Carnitine	32	206	81	94	263	11239	460799
Phosphatidylcholine	188	361	312	343	640	31676	2914192
Sphingomyelin	160	331	189	241	460	21290	319350
Sum	698	2078	1631	3241	4801	205407	3835543
Unique set	399	806	703	768	1246	55952	55952

The number of genes for each metabolite and the corresponding database:interrogation scheme is shown. ^1^ The size of the union of the gene set obtained from all the four database:interrogation schemes. ^2^ The size of the corresponding SNP set. ^3^ The number of tests is the same as the size of the SNP set for the amino acids whereas for aggregated entities like the lipids and carnitine the SNP set is multiplied by the number of compounds present in that class.

### Statistical threshold

The number of unique SNPs generated for each of the metabolites is shown in Table 1. For aggregated metabolites like phosphatidylcholines, sphingomyelins and carnitines the size of the unique SNP set is multiplied by the number of metabolites that fall within each class to yield the total number of tests. For example, the size of the unique SNP set for carnitine is 11,239; this is multiplied by the number of carnitines which is 41, to yield a total number of 460,799 tests for these compounds, as shown in the last column of Table 1. The sum of all SNPs derived from our set of metabolites is 3,835,543. The multiple testing threshold for metabolite concentrations using a Bonferroni correction at a nominal p-value of 0.05 is 1.3E-08 (0.05/3,835,543). In contrast, the p-value threshold for significant association of SNPs with the same metabolite concentrations in the Illig et al. study would be 5.96E-10 (0.05/162*517,840). This represents a reduction of the multiple testing burden by about two orders of magnitude, regardless of the dependency between the SNPs or metabolites.

It has been demonstrated that GWAS of metabolite ratios offer robust statistical associations and point to biological mechanisms related to the interconversion of metabolite pairs. To investigate the association of SNPs with metabolite ratios, we generated the union of SNP sets for all combinations of metabolites (Additional file 1: Table S3). In the case of aggregated metabolites like the lipids and carnitines, the union of the SNP set is multiplied by the number of compounds that fall within each class. For example, the union of the SNP set for arginine and carnitine is 20,000, this is multiplied by 41 to yield the total number of 820,000 tests for this group of ratios. The number of tests for ratios of compounds within classes such as phosphatidylcholines is equal to the size of the unique SNP set multiplied by the number of combinations, n*(n-1)/2, which in this case would be 92*91/2 = 4186. In choosing combinations of ratios, we have assumed that the association p-value for a linear regression model using a metabolite ratio of A/B is equivalent to that computed using it’s reciprocal, B/A. The evidence for lack of independence of a ratio and its reciprocal is provided by the Illig et al. study where a comparison of associations computed using untransformed and log-scaled ratios did not detect significant differences. This implies that we may consider the p-values computed using A/B and B/A to be approximately equal.

The sum of the number of tests for all ratios is 423,645,558 as shown in Additional file 1: Table S3. The multiple testing threshold for the ratios using Bonferroni correction at nominal p-value of 0.05 is 1.18E-10. This represents a multiple threshold reduction by two orders of magnitude over the genome-wide threshold estimated by Illig et al. which is 3.63E-12.

### Proof of principle: sensitivity

The sensitivity of the method was evaluated based on its ability to identify the top hits in the previously published Illig et al. genome-wide association study. The overall sensitivity of the method as well as the interrogation specific breakdown is shown in Table 2. For example, for the BioCyc pathway scheme the size of the unique gene set generated for all the metabolites is shown to be 399. The number of genes that are among the 15 top hits in the Illig et al. study for this database:interrogation scheme is 8 which results in a sensitivity measure of 0.53. A metabolite specific breakdown of each of these schemes and the genes with a p-value cut-off of 1E-02 is shown in Additional file 1: Table S5.

**Table 2:** Performance of the database:interrogation schemes in GWAS dataset analysis

**Database: interrogation scheme**	**Size of gene set**^**1**^	**Top hits from Illig et al. study identified by the method**^**2**^	**Sensitivity**^**3**^
BioCyc pathway	399	*ACADL*, *ACADM*, *ACSL1*, *CPS1*, *FADS1*, *PHGDH*, *SCD*, *SPTLC3*	0.53
BioCyc reaction	806	*ACADM*, *ACADS*, *ACSL1*, *CPS1*, *FADS1*, *SCD*, *SPTLC3*	0.47
KEGG pathway	703	*ACADL*, *ACADM*, *ACADS*, *ACSL1*, *CPS1*, *ELOVL2*, *FADS1*, *PHGDH*, *SCD*, *SPTLC3*	0.67
KEGG reaction	768	*ACADL*, *ACADM*, *ACADS*, *ACSL1*, *CPS1*, *PHGDH*, *SCD*, *SPTLC3*	0.53
Pooled set	1246	*ACADL*, *ACADM*, *ACADS*, *ACSL1*, *CPS1*, *ELOVL2*, *FADS1*, *PHGDH*, *SCD*, *SPTLC3*	0.67

Snapshot of the matches between our method and the association data from the Illig et al. 2010 study for each of the database:interrogation scheme. ^1^corresponds to the unique set of genes generated for all the metabolites for the given database:interrogation scheme. ^2^corresponds to the top hits in the Illig et al. publication that were present in the gene set for the given database:interrogation scheme. ^3^Sensitivity is a measure of the actual positives that have been captured by our method and is equal to the ratio of the number of top hits identified by the method over the total number of top hits in the Illig et al. publication which is 15.

Overall, combining the results from the four database:interrogation schemes helped identify 10 of the 15 top associations (67% sensitivity) published by Illig et al.

### Novel discovery in the Illig et al dataset

Analysis of the first stage or the “discovery stage” dataset of 1029 samples from the Illig et al. dataset yielded several associations with p-values indicative for association, but that did not meet the significance threshold applied by Illig et al. Associations with p-value less than 1E-02 were evaluated in the combined “replication stage” dataset with 1809 samples. Analysis of SNPs in the *ALDH1L1* (aldehyde dehydrogenase family 1 L1) gene locus lowered the p-value of association with serine/glycine ratio from 4.83E-09 in the discovery dataset to 5.13E-12 in the combined dataset. This is well below our threshold of 1.18E-10, but above the threshold to be applied when considering all associations between SNPs and metabolite ratios. Furthermore, the original publication did not select this association for replication because of the threshold set in the first stage of the analysis. This is an example of the method pointing to potential true positives in a genome-wide scan and the association of *ALDH1L1* with the trait is being reported as a novel discovery.

### Statistical threshold in the replication study

The analysis of the Illig et al. dataset identified several biologically relevant candidate genes with p-values less than 1E-02. A list of 56 of these genes associated with phosphatidylcholines and sphingomyelins were investigated in an independent study in the GWAS dataset of phospholipids published by Demirkan et al. The number of matches between the two datasets was: 56 phosphatidylcholines and 6 sphingomyelins. Demirkan et al. also performed GWAS for within class molar proportions for these moieties. We took these into consideration in addition to the GWAS of absolute concentrations. Therefore, the total number of metabolites and proportions investigated in the Demirkan et al. GWAS dataset was 124. A principal component analysis based on the method proposed by Li et al. [16] was performed on this set of metabolites resulting in 51 effectively independent variables. As we considered 2413 independent SNPs in the candidate loci for these metabolites, the statistical threshold, applying Bonferroni correction at a nominal p-value of 0.05, for the replication study was 4.06E-07 (0.05/2413*51).

### Novel discoveries in the replication study

Table 3 shows the top hits in the meta-analysis of candidate genes identified in the Illig et al. dataset for replication. The meta-analysis was performed using Stouffer’s Z-score based method of combining p-values [17]. Since the SNPs in the loci replicated in the Demirkan et al. dataset had relatively low r^2^ values with the SNPs reported in the Illig et al. dataset, we could not perform a traditional meta-analysis where strict linkage disequilibrium criteria are applied. Therefore, we combined the lowest p-value per gene and sought additional supporting evidence for potential allelic heterogeneity (see Discussion). As mentioned earlier, the p-value threshold for the replication study is set at 4.06E-07. SNPs in the vicinity of the genes *CBS, GPAM, ADCY8*, *CNR1*, *HSD17B12*, *MBOAT1*, *PECR*, *PLCB1* and *TECR* pass this threshold.

**Table 3:** Replication of candidate genes in the Demirkan et al. dataset

**Gene**	**Trait**	**SNP from the Illig et al. dataset**	**p-value**^**1**^	**SNP from the Demirkan et al. dataset**	**p-value**^**2**^	**Combined p-value**^**3**^
*ADCY8*	PC ae C40:6	rs11786743	4.03E-05	rs913819	6.73E-04	2.15E-07
CBS^*^	PC ae C40:6	rs2839631	5.67E-06	rs378376	5.17E-04	2.90E-08
*CNR1*	PC ae C38:2	rs10485168	2.42E-04	rs9359765	4.61E-04	7.54E-07
GPAM^*^	PC ae C34:3	rs2246253	1.25E-04	rs2419603	1.76E-04	1.56E-07
*HSD17B12*	PC aa C34:4	rs2862999	2.66E-05	rs11037685	6.13E-04	1.35E-07
*MBOAT1*	PC ae C40:6	rs9465673	1.11E-04	rs694094	4.47E-04	3.53E-07
*PECR*	PC aa C38:0	rs3770536	5.55E-04	rs3770562	9.43E-05	3.79E-07
*PLCB1*	PC aa C30:0	rs6056188	9.55E-06	rs17363114	1.96E-03	2.06E-07
*TECR*	PC aa C32:0	rs7252966	1.69E-05	rs7254215	2.09E-03	3.57E-07

Top hits from the meta-analysis of candidate genes identified in the Illig et al. study and replicated in the Demirkan et al. dataset. ^1,2,3^p-value of association of the SNP with the trait in the Illig et al., Demirkan et al. and combined p-value respectively. ^*^indicates genes for which further evidence was found.

## Discussion

Genome wide association studies with metabolites as phenotypes have identified several loci that explain human metabolic individuality. However, the large metabolite panel being tested results in a severe multiple testing burden that precludes genuine SNP-metabolite pairs from consideration when they fail to reach the stringent threshold for statistical significance. Our method aims to address this problem by selectively testing genes that operate in reactions and pathways relevant to the metabolite. The goal is to reduce the severity of the multiple testing burden and identify potential true positives in the list of genome-wide associations. Taverna, a workflow management system was used to generate the SNP-metabolite pairs. We have deposited the workflows at a repository called myexperiment.org, making it easier for the scientific community to interpret, repeat and reproduce the result. The sensitivity of the method, defined as retrieval of previously identified associations, is high, as evident from the proof of principle study carried out on the genome scan published by Illig et al. Replication studies on some of the promising SNP-metabolite pairs identified by the method pointed to a novel and statistically significant association at the *ALDH1L1* locus with serine/glycine ratios. Additional replication studies of phosphatidylcholines and sphingomyelins uncovered significant gene-wise associations with *CBS, GPAM, ADCY8*, *CNR1*, *HSD17B12*, *MBOAT1*, *PECR*, *PLCB1* and *TECR.*

### Databases, interrogation schemes and software tool

The pathway databases have technical and conceptual differences [11] that mandate interrogation of multiple databases and integration of the results. Interpretation of these results requires a close coordination between biologists and computer scientists. Workflow management systems in general and Taverna [Additional file 1: S2] in particular is an example of a software tool that is intuitive enough for the biologist, while at the same time offering the flexibility for exploring the algorithmic aspects for the computer scientist [18]. In using Taverna as a software tool and depositing the workflows in the repository myexperiment.org, we have attempted to make the method and the rationale transparent to users, thus facilitating its retrieval, reuse and reproduction by other independent scientists [19].

### Sensitivity of the method

As a sensitivity measure of our method, we evaluated its ability to pick the top hits in the Illig et al. publication [2]. Some 60% of the top associations were identified successfully. A similar analysis of GWAS dataset published by Suhre et al. [3] yielded a sensitivity of 54% (20 out of 37 hits) (data not shown). However, 4 of the “misses” in the Suhre et al. dataset were peptide fragments that do not have an entry in the pathway databases, which is a prerequisite for our method to work.

We interpret the high sensitivity of our method in three ways; first it reinforces the rationale that GWAS with metabolomic phenotypes provides a functional approach to the study of human genetic variation [1]. In other words, the known function of the associated gene and the biochemical characteristics of the affected metabolite support each other in ways that lends itself to a narrative on the underlying biological mechanism. Second, while the pathway databases have a long way to go in achieving a comprehensive annotation and delineation of biological processes, they, however, are a good resource of information in so far as the top hits in a GWAS with metabolomic phenotypes are concerned. Only two out of the 15 top hits in the study by Illig et al. were genes with unknown functions (*PLEKHH1*, *SYNE2*), and two others were hitherto uncharacterized solute transporters (*SLC16A9*, *SLC22A4*). Third, a good sensitivity measure is a validation of our method and reflects its comprehensive data collection ability through integration of disparate data sources and utilization of appropriate interrogation strategies.

### Novel discoveries

**Figure 4:**
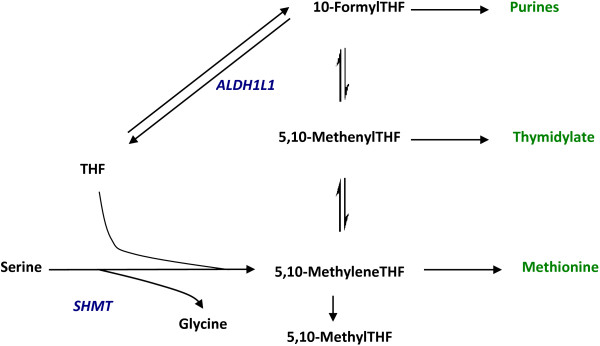
**Role of ALDH1L1 in the cytosolic one-carbon pool metabolism.** A simplified schematic of the one-carbon pool metabolism in the cytosol is depicted. *ALDH1L1*: Aldehyde Dehydrogenase 1 Family, Member L1; *THF*: tetrahydrofolate; *SHMT*: Serine hydrxymethyltransferase.

Our analysis of the GWAS dataset of the Illig et al. publication based on the first step of the “discovery design” yielded several interesting associations that had not been reported among the top hits in the publication. We selected a few of the promising associations for replication in the combined dataset of 1809 subjects. One of the genes, Aldehyde dehydrogenase family 1 L1 (*ALDH1L1*) was found associated with the ratio of serine/glycine with a p-value of 5.13E-12 in the combined set of 1809 subjects. *ALDH1L1* also known as 10-formyltetrahydrofolate dehydrogenase (*10-FTHFDH*, *FDH*) catalyzes the NADP^+^ dependent oxidation of 10-formyltetrahydrofolate to CO_2_ and tetrahydrofolate (THF) [20] as shown in Figure 4. It plays an important role in folate metabolism [21-25]. Among other functions, *ALDH1L1* has been known to deplete cellular 10-formyltetrahydrofolate pool resulting in a loss of *de novo* purine biosynthesis [23], maintain cellular folate concentrations by regulating the availability of THF [22], but most importantly, it has been shown to compete with the enzyme serine hydroxymethyl transferase (*SHMT*) for the polyglutamyltetrahydrofolates [25]. The latter enzyme catalyzes the conversion of serine to glycine as shown in Figure 4. It has also been shown that glycine to serine inter-conversion by *SHMT* accounts for approximately 41% of whole body glycine flux inclusive of both mitochondrial and cytoplasmic processes [26].

To further investigate the potential of our approach to uncover novel genetic associations, we extended the analysis to an additional independent GWAS dataset [4]. Candidate genes identified in the Illig et al. dataset in association with phosphatidylcholines and sphingomyelins were considered for replication in the dataset provided by Demirkan et al. [4]. We discuss here two novel findings for which additional evidence was obtained.

SNPs near glycerol-3 phosphate acyltransferase (*GPAM)* are associated with PC ae C34:3 moieties in the Illig et al. and Demirkan et al. datasets with p-values of 1.25E-04 and 1.75E-04, respectively, with a meta-analysis p-value of 1.56E-07. *GPAM* encodes a mitochondrial protein that esterifies the acyl group from acyl-coA to the sn-1 position of glycerol-3-phosphate. It is a rate-limiting enzyme that catalyzes the initial step in the biosynthesis of triacylglycerols and phospholipids [27]. A recent study showed that in breast cancer, *GPAM* expression is strongly correlated with survival rates, clinico-pathological features as well as metabolomic and lipidomic profiles [28]. Interestingly, the study identified the metabolite PC C34:3 as the most significantly altered metabolite with respect to GPAM expression in breast cancer patients. This suggests that, for this particular example, genetic control is primarily at the level of gene expression, with secondary effects on enzyme levels and metabolic conversion rates. The example also highlights the potential influence of genetic variation of metabolic pathways on disease.

A large number of genes identified by our method in the context of phospholipids participate in fatty acid metabolism and are therefore likely to affect the levels of groups of phosphatidylcholines and sphingomyelins. For example, *GPAM* esterifies the acyl group from acyl-ACP to the sn-1 position of glycerol-3-phosphate, and is therefore relevant to both acyl-acyl and acyl-alkyl moieties. The lowest p-value of association, at this locus, with a phosphatidylcholine moiety in the Illig et al. study is with PC ae C36:3, while in the Demirkan et al. study it is PC aa C36:3. Since both associations make biological sense, future work should incorporate joint modelling of suitable phospholipid moieties to help identify loci that are biologically relevant but fail to reach the statistical threshold in GWAS analysis. We have reported such best case associations for phosphatidylcholines in Additional file 1: Table S6.

SNPs near Cystathionine beta-synthase (*CBS)* are associated with PC ae C40:6 moieties in the Illig et al. and Demirkan et al. datasets with p-values of 5.67E-06 and 5.17E-04, respectively, with a meta-analysis p-value of 2.9E-08. Mutations in *CBS* cause hyperhomocysteinemia [29], which is marked by elevated levels of homocysteine. Several studies have associated altered phosphatidylcholine biosynthesis with hyperhomocysteinemia/*CBS* deficiency [30-33]. In one of the studies [30], phosphatidylcholine levels and the activity of the enzyme lecithin-cholesterol acyltransferase (*LCAT*) were significantly lower in *CBS* deficient mice than in wild type mice. While there is considerable literature evidence for the role of *CBS* in phosphatidylcholine metabolism, the stringent p-value threshold obscures this association in the list of GWAS results.

The low r^2^ values for significant SNPs in *GPAM*, *CBS* and other loci between the Illig et al. and Demirkan et al. datasets could be explained by allelic heterogeneity. The latter is a phenomenon where multiple alleles from one gene influence a trait. However, in some cases it may be that the two apparently independent SNPs are tagging a third SNP [34]. This may be the case for the two SNPs (rs2839631, rs378376) near *CBS* which have an r^2^ of 0.067 and are associated with C40:6 phosphatidylcholines in both the datasets. However, both SNPs are in LD with *cis*-eQTLs in the region (for example, rs719037, r^2^ ~ 0.4). This is suggestive of the SNPs exerting their effect through the expression levels of the *CBS* enzyme, as was suggested for *GPAM*. Apparent allelic heterogeneity may preclude identification in a standard meta-analysis, but would justify further investigation of independent or dependent signals at loci showing this phenomenon.

### Challenges and future direction

In general, our effort was directed at exploring the utility of machine-enabled interrogation of metabolic pathway databases in prioritizing SNP-metabolite associations in a GWAS dataset. While the method’s sensitivity and ability to make novel discovery are encouraging, considerable progress needs to be made in metabolite disambiguation to achieve a relevant and comprehensive gene set for a given metabolite. This problem is particularly acute for phospholipids like phosphatidylcholines and sphingomyelins and various forms of the fatty acid transporters of L-carnitine. For example, the metabolomics technology used in the Illig et al. study differentiated more than 90 forms of phosphatidylcholines based on alkyl or acyl bonds and single or double bonds on the side chains. However, the pathway databases do not yet contain information for the complex structures. This forces users to analyze these metabolites at a higher aggregation level. Another issue that requires attention is the bias introduced in selecting genes for inclusion in the gene set. We have formulated simple rules for interrogation [Additional file 1: S1] that facilitates unbiased generation of gene sets for any given metabolite.

Another challenge arises due to the high correlation between metabolites, particularly the phospholipids like phosphatidylcholines and sphingomyelins. These moieties are associated with loci relevant to fatty acid metabolism. While the variation at these loci effects the levels of fatty acids and thereby the phospholipid pool, to a large extent, these loci are not specific for any particular phospholipid moiety. As a result, several loci exhibit a pleiotropic effect for biologically related metabolic phenotypes in general and phospholipids in particular [Shown in Additional file 1: Table S7] We have demonstrated that background knowledge and evidence-based approach is ideally suited to identify such candidate genes, however future work should focus on statistical methodologies with sufficient power to detect such pleiotropic loci in GWAS of intermediate phenotypes. In summary, future work includes integration of more pathway databases, metabolite disambiguation, consideration of allelic heterogeneity and multivariate statistical techniques that take into account the high degree of correlation between the metabolites.

## Conclusions

A measurement of metabolites as intermediate phenotypes is a potentially powerful approach to uncover the influence of genetic variation on disease susceptibility and progression. However, we still face many hurdles in the interpretation of GWAS data. In this study, we investigated the utility of background knowledge present in pathway databases in extending our understanding of the genetic basis of metabolic variation. We developed a bioinformatics method that prioritizes SNP-metabolite associations in a GWAS based on metabolic pathway information present in the KEGG and BioCyc databases. The validity of the method is demonstrated by re-analysing published GWAS datasets and identifying previously known associations. We report a new locus of human metabolic individuality, *ALDH1L1* (Aldehyde dehydrogenase family 1 L1) associated with serine/glycine ratios. Replication studies in an independent GWAS of phospholipids identified *GPAM* (Glycerol-3 phosphate acyltransferase) and *CBS* (Cystathionine beta-synthase) as novel loci, and this was further supported by additional literature evidence. The utility of a workflow management system in facilitating novel biological discoveries and as a tool for efficient sharing of computational protocols is demonstrated.

## Methods

### GWAS data set for proof of principle studies

The GWAS dataset published by Illig et al. 2010 [2] was used to evaluate the validity of the method. Illig et al. employed a two-stage discovery design in the KORA F4 population cohort with 1029 male and female individuals in the first stage and 780 individuals in the second stage. Loci with p-value of association <10^-7^ for metabolite concentrations and p-value < 10^-9^ for concentration ratios were taken up for the second stage independent testing in 780 individuals. The joint p-values of association for all the 1809 individuals were then computed and 15 loci were reported whose strength of association increased after the second stage of the discovery process. The authors note that “although this approach is less well powered than a full genome-wide joint analysis, it reflects the historical way in which [they] selected SNPs for follow-up”. This means that if we can identify potential true positives using the 1029 samples, we can validate them in the full dataset, since this has not been done in the Illig et al. study for all hits with p-value > 10^-7^ for metabolite concentrations and p-value > 10^-9^ for concentration ratios. Therefore, the GWAS dataset based on 1029 samples was analyzed for our proof of principle studies. Additionally, to evaluate novel associations identified by the method in the discovery stage dataset, the strength of the signal was assessed in the combined GWAS dataset for 1809 subjects.

### GWAS dataset for follow-up studies

Candidate loci identified in the Illig et al. dataset by our method were taken up for follow-up studies in the dataset published by Demirkan et al. The latter conducted a meta-analysis of GWAS on plasma levels of ceramides, phosphatidylcholines, lysophosphatidylcholines, sphingomyelins, phosphatidylethanolamines and plasmalogens in five European populations: the Erasmus Rucphen Family (ERF) study, conducted in the Netherlands, (2) the MICROS study from the Tyrol region in Italy, (3) the Northern Swedish Population Health Survey (NSPHS) in

Norrbotten, Sweden, (4) the Orkney Complex Disease Study (ORCADES) in Scotland, and (5) the CROAS (CROATIA_Vis) study conducted on Vis Island, Croatia. Broadly, the metabolite overlap between the Illig et al. dataset and Demirkan A et al. dataset was confined to the class of phosphatidylcholines, lysophosphatidylcholines and sphingomyelins. More specifically, the overlap represented 62 phospholipid moieties. Also, 56 candidate genes were identified for follow up in the Illig et al. dataset. We choose to focus on SNPs in the flanking 50 kb region of these genes for the follow-up study in the Demirkan A et al. dataset.

### Metabolites considered for the generation of gene sets

Gene sets are defined as entities that participate in pathways and reactions relevant to the metabolite and hence hold the potential to influence its levels. The goal was to generate gene sets for the compounds that were measured in the Illig et al. 2010 publication: 14 amino acids (Arginine, Glutamine, Glycine, Histidine, Methionine, Ornithine, Phenylalanine, Proline, Serine, Threonine, Tryptophan, Tyrosine, Valine, and Leucine), 41 Carnitines, 92 Phosphatidylcholines and 15 Sphingomyelins. In addition to the metabolites mentioned above, Illig et al. also measured Hexose. We did not consider this metabolite for investigation because pathway information surrounding hexose is lacking. While metabolites like glucose and fructose could have been considered as proxies, we did not pursue this because of the enormous size of the resulting gene set, combined with a lack of confidence in the relevance of many of these genes to the metabolite measured by the metabolomics platform.

### Pathway databases and interrogation schemes

The metabolic pathway databases KEGG (release 63) [12] and BioCyc (version 16) [13] were accessed for retrieving background knowledge surrounding metabolites. Two interrogation schemes were employed: pathway scheme and reaction scheme (Figure 1). In a pathway scheme, for a given metabolite, all the pathways that it participates in are determined followed by the retrieval of all the genes that participate in these pathways (Figure 1A). In a reaction scheme, given a metabolite, all the reactions that it is part of and the compounds that participate in these reactions are determined. The compounds obtained at this point are subjected to the same strategy as in the previous step in that all the reactions that these compounds participate in are determined. This can be visualized as expanding by a radius of 2 steps in the reaction space of every metabolite. Finally, the enzymes that drive all these reactions are determined (Figure 1B). As an intermediate step certain compounds were filtered out in order to avoid non-specific connections. The details about the filtration step and the compounds that were filtered are provided in the Additional file 1: S4. In all there are four schemes: kegg:pathway, kegg:reaction, biocyc:pathway, and biocyc:reaction. The set of non-redundant genes combined from all the schemes then forms the gene set for any given metabolite.

### Software used to generate gene and SNP sets

Taverna version 2.4 [14], a workflow management system was used to generate metabolite specific gene sets as well as for the generation of SNPs present in the 50 kb flanking region of each gene. Taverna allows users access to remote data resources like KEGG, BioCyc, Ensembl, NCBI etc. and data management systems like Biomart through implementation of web services. Each component in a workflow is responsible for a particular function and many such components need to be chained together in a pipeline to create a workflow that performs a certain task. The pipeline depicted in Figure 1 is implemented in a Taverna workflow through appropriate linking of remote web services and local scripts. Web services are software systems that facilitate machine to machine interaction over a network. Taverna allows the inclusion of different kinds of web services like Web Services Description Language (WSDL) and REpresentational State Transfer (REST). The services provided by the KEGG database were implemented using the REST services made available in the Taverna workbench. The BioCyc database was accessed through the REST interface using the BioVelo language. The latter is a query language designed to let the users write precise queries against the pathway/genome databases, available at BioCyc, to retrieve pathways, reactions, compounds, genes etc. All the workflows were designed following best practices for workflow design [35].

### Workflow accessibility

To facilitate retrieval and reproducibility, the workflows have been deposited in a repository at http://www.myexperiment.org/packs/319.html. While the focus of this paper was on a specific set of metabolites; using appropriate identifiers from the KEGG or BioCyc database users will be able to generate gene sets for other metabolites. To generate a gene set for any metabolite using the KEGG or BioCyc database, users have to input the metabolite identifier for that database and the output is a text file containing the entrez gene identifiers. For example, to generate a gene set for the metabolite Arginine, for either the pathway or reaction scheme using the KEGG database, users input the KEGG identifier for Arginine: C00062. Similarly, to obtain a gene set using the BioCyc database, the input for the same metabolite is “L-arginine”. The workflows may also be repurposed to suit other objectives, for example, to filter out non-specific connections, we remove hub metabolites like ATP, NADP and other entities like co-enzymes; however, users may change the filtration criteria if they find it too stringent for their objectives. A detailed tutorial on how to access and run these workflows is provided in the Additional file 1: S2.

## Competing interests

The authors declare that they have no competing interests.

## Authors’ contributions

HD created the workflows and the algorithms to analyze GWAS datasets. HD, PH, KWvD, and PACtH prepared the manuscript. PH, KWvD and PACtH contributed ideas and supervised the project. JK contributed towards the analysis of GWAS datasets. MR, KH assisted in design of workflows. DMK, CG, RWG, JA, KS, AD, CMD provided the data sets for this study. All authors read and approved the final manuscript.

## Supplementary Material

Additional file 1 **S1.** Rules to generate Metabolite-Gene sets. **S2. **Taverna workflow management system. **Figure S1.** Snapshot of the Taverna workbench which consists of three panels as pointed to in the figure. **Table S3.** SNP set generated for ratios of metabolites. **S4.** Compounds filtered for the Kegg:Reaction Scheme. **Table S5.** Metabolite specific break-up of the performance of database:interrogation schemes. **Table S6.** Best case associations of loci with phosphatidylcholines in the Illig *et al* and Demirkan *et al* datasets. **Table S7.** Pleiotropic effect for phosphatidylcholines at select loci. Click here for file
