# Supplementary material for: Automated workflow-based exploitation of pathway databases provides new insights into genetic associations of metabolite profiles
Source: BMC Genomics. 2013 Dec 9;14:865. doi: 10.1186/1471-2164-14-865 (PMC3879060; doi:10.1186/1471-2164-14-865)
Supplement: Additional file 1 — S1. Rules to generate Metabolite-Gene sets. S2. Taverna workflow management system. Figure S1. Snapshot of the Taverna workbench which consists of three panels as pointed to in the figure. Table S3. SNP set generated for ratios of metabolites. S4. Compounds filtered for the Kegg:Reaction Scheme. Table S5. Metabolite specific break-up of the performance of database:interrogation schemes. Table S6. Best case associations of loci with phosphatidylcholines in the Illig et al and Demirkan et al datasets. Table S7. Pleiotropic effect for phosphatidylcholines at select loci. [file 1471-2164-14-865-S1.doc]

**Additional file 1: S1. Rules to generate Metabolite-Gene sets:**

1. Metabolic pathway databases accessed via Taverna workflows: KEGG, Biocyc
2. Interrogation Scheme:
   1. Pathway Scheme (KEGG:Pathway and Biocyc:Pathway): Given a metabolite determine all the pathways it participates in and pull all the genes that participate in these pathways (Fig 2A).
      1. For KEGG consider only metabolic pathways.
      2. Phosphatidylcholine and sphingomyelins contain fatty acids in their side chains. Genes that are involved in fatty acid metabolism alter the levels of phospholipids. Previously published GWAS datasets have shown that most of the significant genes associated with phospholipids, for example *FADS1,* are involved in fatty acid metabolism. To incorporate such genes we ran the pathway and the reaction scheme on various fatty acids and incorporated them into the gene set for phosphatidylcholines and sphingomyelins. For the biocyc:pathway scheme, gene set for phosphatidylcholine and sphingomyelin contained genes generated for: arachidonate, a fatty acid, laurate, linoleate, a lipid, a long chain fatty acid, octanoate, oleate, palmitate, a 2,3,4 saturated fatty acid and stearate. Similarly, for the kegg:pathway scheme the gene sets for the following compounds were included in the set for phosphatidylcholine and sphingomyelin: arachidonic acid (C00219) and palmitic acid (C00249). The choice of fatty acids was based on whether pathway information was available for the compounds for the database being considered.
      3. Biocyc:Pathway for carnitines included the following compounds: L-Carnitines, Palmitoylcarnitine, L-Ocatnoylcarnitine, O-acetylcarnitine, butanoyl-CoA, decanoyl-CoA, lauroyl-CoA, myristoyl-CoA,octanoyl-CoA, palmitoyl-CoA, and stearoyl-CoA. The rationale behind this inclusion is provided below in the “Reaction Scheme”.
   2. Reaction Scheme (KEGG:Reaction and Biocyc:Reaction): Given a compound find all the reactions and the compounds that participate in these reactions. The reactions that these compounds participate in and the enzymes that drive these reactions are determined (Fig 2B).
      1. Compounds that make too general connections are filtered out. List of filtered compounds for Kegg and Biocyc database interrogation provided.
      2. For the biocyc:reaction scheme gene sets for the following compounds were included in the set for phosphatidylcholine and sphingomyelin: (9Z)-12,13-dihydroxyoctadeca-9-enoate, octanoate, laurate, decanoate, stearate, palmitate, oleate, myristate, linoleate, arachidonate, arachidate, a long-chain fatty acid, a fatty acid, a phospholipid, and a lipid. For the kegg:reaction scheme the following fatty acids were included for the purpose of generating a gene set for phosphatidylcholines and sphingomyelins: arachidonic acid (C00219), linoleic acid (C01595), palmitic acid (C00249) and stearic acid (C01530). The choice of fatty acids was based on whether reaction information was available for the compounds for the database being considered.
      3. Biocyc compounds are structured as classes. We have established the following rules for interrogation:
         1. In general for amino acids we do not consider the super class and we don’t have to deal with child terms.
         2. Phosphatidylcholine and Sphingomyelin, are considered as “class” terms. Sphingomyelin does not have child terms, phosphatidylcholine does have child terms but the reactions in the database are not considered at the level of the latter. For both phosphatidylcholine and sphingomyelin the parent terms (a phosphoglyceride and a sphingolipid respectively) are not considered for interrogation.
         3. For carnitines, we combine results for the L-carnitine, L-Octanoylcarnitine,L-palmitoylcarnitine, O-acetylcarnitine (these are the only carnitines present in the database, there is considerable overlap in terms of genes returned for the four compounds). Two of the compounds returned for these interrogations: palmitoyl-CoA and octanoyl-CoA are instances of “a 2,3,4 saturated fatty acid” and since some of the reactions are given at the level of the parent class, we have also included “a 2,3,4 saturated fatty acid” in the Biocyc:Reaction interrogation scheme.
            - The various instances of “a 2,3,4 saturated fatty acid” are: butanoyl-CoA,decanoyl-CoA,lauroyl-CoA,myristoyl-CoA,octanoyl-CoA,palmitoyl-CoA,stearoyl-CoA. These are all the acyl fatty acids that are transported by carnitine for mitochondrial fatty acid beta oxidation. The corresponding esters were all measured by Illig et al, therefore it was decided to consider all the above instances of “a 2,3,4 saturated fatty acid” to generate the gene set for Carnitine.

In a nutshell then, we have four schemes that yield genes that operate in the vicinity of a given metabolite: Kegg:Pathway, Kegg:Reaction, Biocyc:Pathway, Biocyc:Reaction. As an example, Kegg:Pathway means employing the pathway scheme as mentioned above on the Kegg database. Table 1 displays the yield of genes for each database:interrogation scheme. The gene set for a metabolite is the integration of all the genes coming out of the four schemes into a non-redundant set as shown in the last column of Table 1 in the publication. The sum of all such non-redundant set equals 4801 genes. The total number of unique genes that came out of all the schemes and all the metabolites is 1246 with the number of unique genes from Kegg being 379 and those from Biocyc being 227 and 640 genes present in both databases (Fig 3 of the publication).

**Additional file 1: S2. Taverna workflow management system**

Workflow management system is a software environment designed to compose and execute a series of computational or data manipulation steps. Taverna workbench is an example of a workflow management system that provides a desktop environment for accomplishing bioinformatic tasks. Taverna allows users access to data sources and analysis tools made available by institutions like NCBI, DDBJ, EBI etc through web services. In addition to making available third-party services, Taverna offers a suite of shim services that run on the local computer and are essentially used for data manipulation.

A Taverna workflow is a directed acyclic graph consisting of components (web or shim services) having various functionalities chained together appropriately to perform a useful task. Figure 1 shows a snapshot of the Taverna workbench that consists of three panels: a service panel at the top left that makes available third-party services and also a few local services that are included by default, the panel on the right showing the workflow diagram is a space where workflows can be created by pulling services from the left panel in a drag and drop fashion. Existing workflows can be opened in the workflow canvas using the open tab. Later in this section, a tutorial on how to open workflows stored on myExperiments.org is provided. The panel in the bottom left is known as the workflow explorer which depicts the workflow in a tree like fashion and allows editing of properties of the components of the workflow.

**Downloading the Taverna workflow management system**

Instructions for downloading Taverna can be found at: <http://www.taverna.org.uk/download/>

The tutorials to learn about features and how to run Taverna are available at the Taverna web site: <http://www.taverna.org.uk/documentation/taverna-2-x/quick-start-guide/>

**
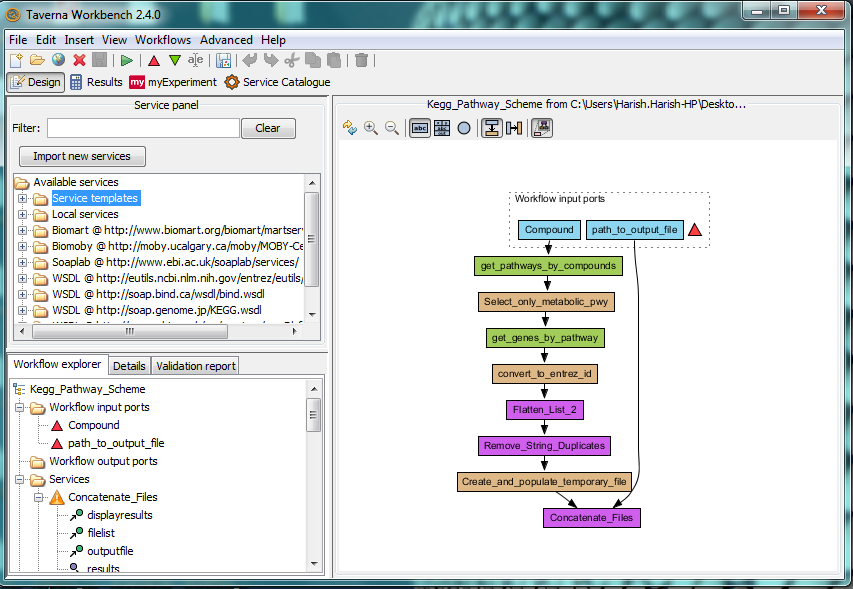
**

**Service panel**

**Workflow canvas**

**Information panel**

**Additional file 1: Figure S1** Snapshot of the Taverna workbench which consists of three panels as pointed to in the figure.

**Tutorial: Download workflow from myExperiment.org, learn more about workflow functionality and run the workflows**

**
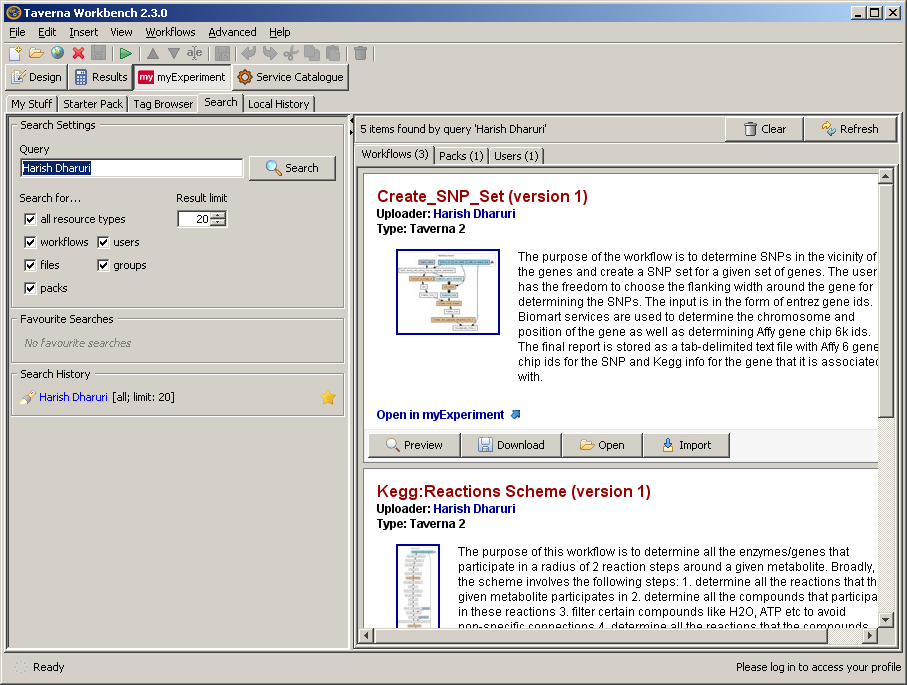
**

1. Click on myExperiment tab 🡪 followed by the ‘Search’ button.
2. Type in the name ‘Harish Dharuri’ in the query and click the Search button
3. This should display the models that we have submitted to myExperiment in the right panel.
4. Choose the model that you would like to run and click the ‘Open’ button.


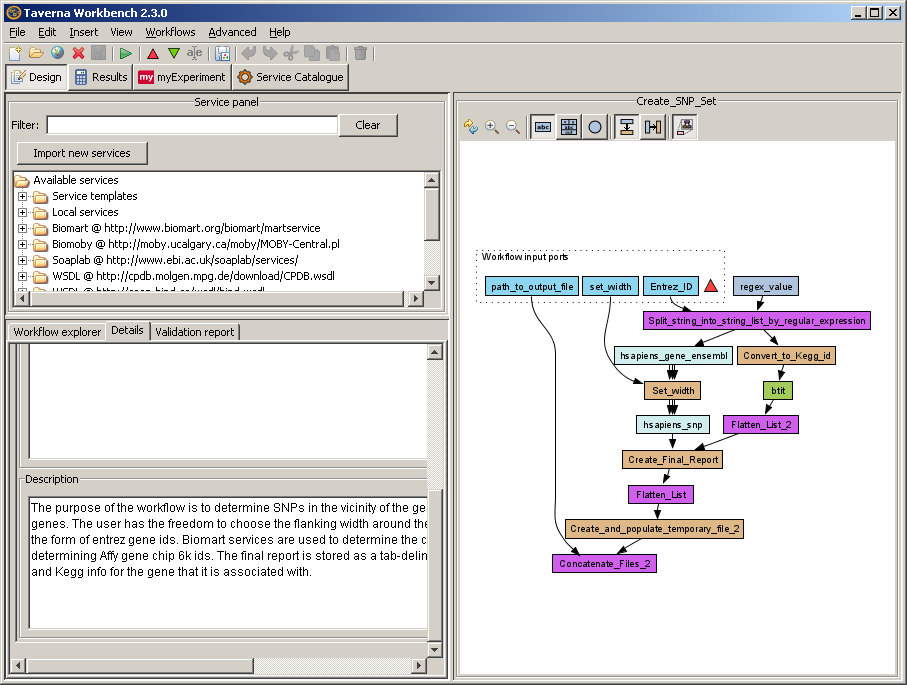


**Details**

1. This will open the workflow in the ‘Design’ mode of Taverna.
2. Click the ‘Details’ to read the description of the workflow.
3. Click on any input ports and read the annotation in ‘Details’ to know about input type required.
4. Run the workflow by clicking this button

**
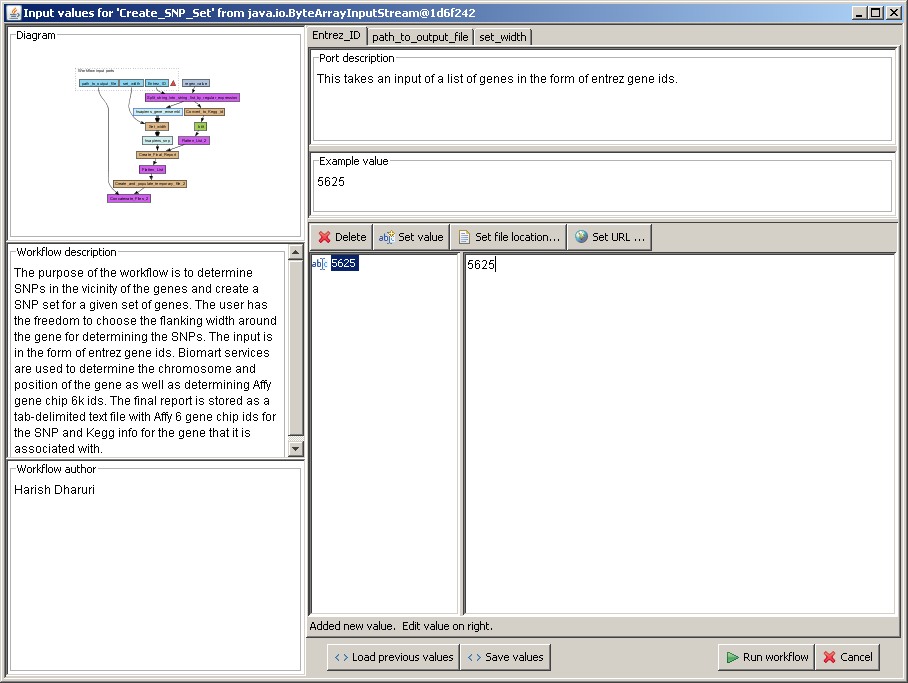
**

1. This will open the ‘input dialog’ to enter the input values.
2. Click on the tabs at the top-right of the panel for each of input to know the example value.
3. Click ‘Set value’ and change/enter a value.
4. Press the ‘Run workflow’ button when you are done entering all the values.

**
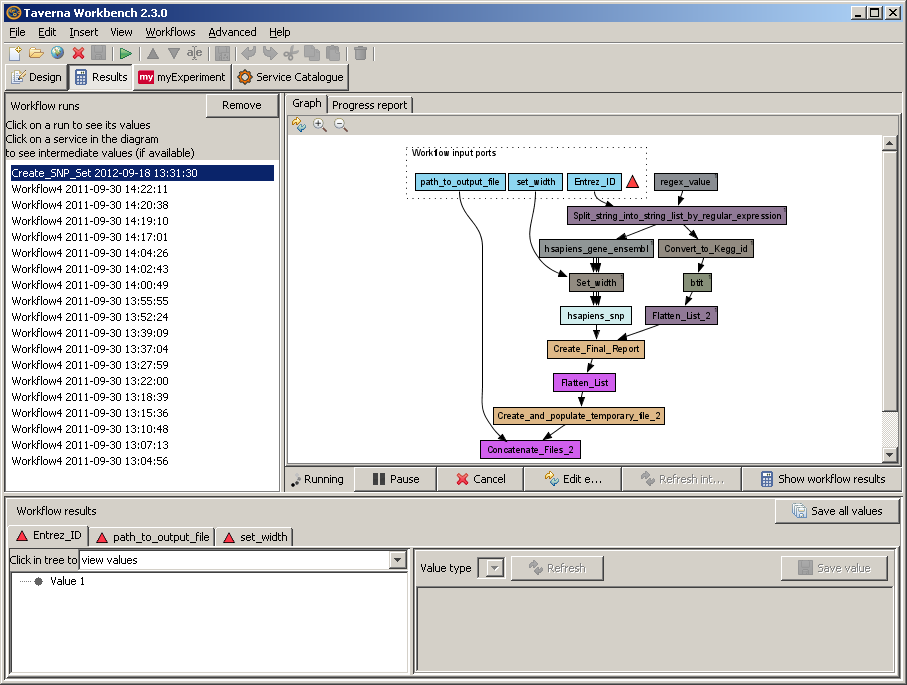
**

1. This will open the workflow in the ‘Result’ mode of Taverna.
2. Watch the progress of your run in the default ‘Graph’ mode as shown in the picture on the right.

**
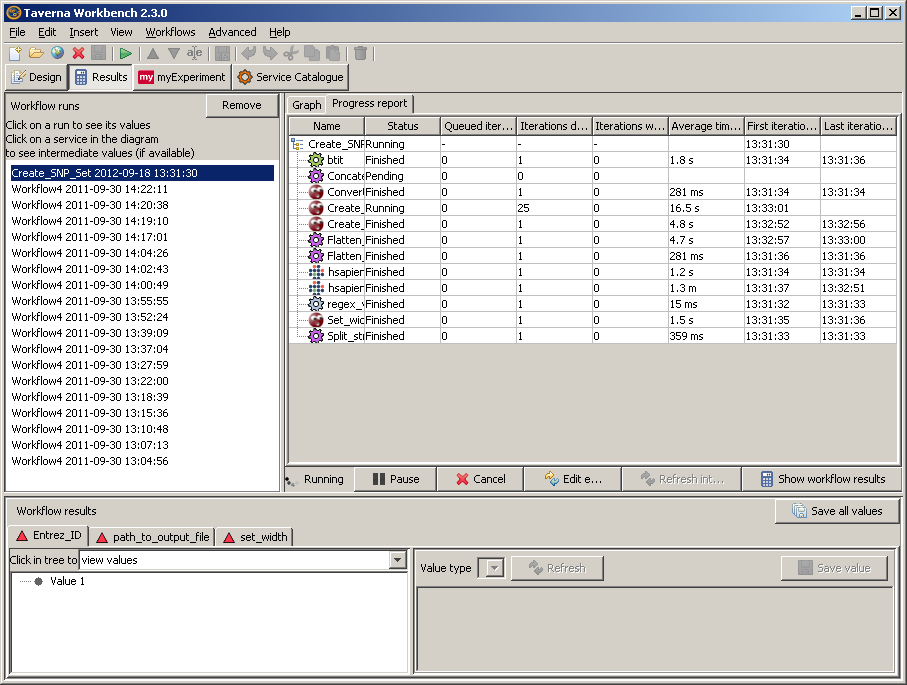
**

1. Or click the ‘Progress Report’ tab to see the progress of the run in tabular mode of the Result mode as shown in the picture at the right.
2. At the end of the run the results will be stored as a text file in a path provided as input by the user.

**Alternate way: The workflows may be downloaded from:** <http://www.myexperiment.org/packs/319.html> and run in Taverna.

**Additional file 1: Table S3. SNP set generated for ratios of metabolites**

| **Metabolite Ratio** | **Union Set^1^** | **Number of tests^2^** |
| --- | --- | --- |
| Arginine/Carnitine | 20000 | 820000 |
| Arginine/Glutamine | 20340 | 20340 |
| Arginine/Glycine | 26234 | 26234 |
| Arginine/Histidine | 14743 | 14743 |
| Arginine/Leucine | 13133 | 13133 |
| Arginine/Methionine | 18982 | 18982 |
| Arginine/Ornithine | 14049 | 14049 |
| Arginine/Phenylalanine | 14577 | 14577 |
| Arginine/Phosphatidylcholine | 44228 | 4068976 |
| Arginine/Proline | 11897 | 11897 |
| Arginine/Serine | 23269 | 23269 |
| Arginine/Sphingomyelin | 33739 | 506085 |
| Arginine/Threonine | 12362 | 12362 |
| Arginine/Tryptophan | 16681 | 16681 |
| Arginine/Tyrosine | 15865 | 15865 |
| Arginine/Valine | 15865 | 15865 |
| Carnitine/Glutamine | 23428 | 960548 |
| Carnitine/Glycine | 27500 | 1127500 |
| Carnitine/Histidine | 16172 | 663052 |
| Carnitine/Leucine | 13595 | 557395 |
| Carnitine/Methionine | 20612 | 845092 |
| Carnitine/Ornithine | 19817 | 812497 |
| Carnitine/Phenylalanine | 17971 | 736811 |
| Carnitine/Phosphatidylcholine | 37658 | 142045976 |
| Carnitine/Proline | 14282 | 585562 |
| Carnitine/Serine | 23453 | 961573 |
| Carnitine/Sphingomyelin | 27892 | 17153580 |
| Carnitine/Threonine | 13388 | 548908 |
| Carnitine/Tryptophan | 19031 | 780271 |
| Carnitine/Tyrosine | 17279 | 708439 |
| Carnitine/Valine | 17279 | 708439 |
| Glutamine/Glycine | 28589 | 28589 |
| Glutamine/Histidine | 19521 | 19521 |
| Glutamine/Leucine | 17644 | 17644 |
| Glutamine/Methionine | 23790 | 23790 |
| Glutamine/Ornithine | 19818 | 19818 |
| Glutamine/Phenylalanine | 17948 | 17948 |
| Glutamine/Phosphatidylcholine | 46176 | 4248192 |
| Glutamine/Proline | 17411 | 17411 |
| Glutamine/Serine | 27052 | 27052 |
| Glutamine/Sphingomyelin | 35706 | 535590 |
| Glutamine/Threonine | 17943 | 17943 |
| Glutamine/Tryptophan | 21007 | 21007 |
| Glutamine/Tyrosine | 19595 | 19595 |
| Glutamine/Valine | 19595 | 19595 |
| Glycine/Histidine | 23648 | 23648 |
| Glycine/Leucine | 23462 | 23462 |
| Glycine/Methionine | 24339 | 24339 |
| Glycine/Ornithine | 24918 | 24918 |
| Glycine/Phenylalanine | 25261 | 25261 |
| Glycine/Phosphatidylcholine | 46133 | 4244236 |
| Glycine/Proline | 23758 | 23758 |
| Glycine/Serine | 28932 | 28932 |
| Glycine/Sphingomyelin | 38072 | 571080 |
| Glycine/Threonine | 22578 | 22578 |
| Glycine/Tryptophan | 24651 | 24651 |
| Glycine/Tyrosine | 25633 | 25633 |
| Glycine/Valine | 25633 | 25633 |
| Histidine/Leucine | 8881 | 8881 |
| Histidine/Methionine | 13622 | 13622 |
| Histidine/Ornithine | 14270 | 14270 |
| Histidine/Phenylalanine | 12238 | 12238 |
| Histidine/Phosphatidylcholine | 38491 | 3541172 |
| Histidine/Proline | 9457 | 9457 |
| Histidine/Serine | 21504 | 21504 |
| Histidine/Sphingomyelin | 29802 | 447030 |
| Histidine/Threonine | 9257 | 9257 |
| Histidine/Tryptophan | 12229 | 12229 |
| Histidine/Tyrosine | 13034 | 13034 |
| Histidine/Valine | 13034 | 13034 |
| Leucine/Methionine | 13718 | 13718 |
| Leucine/Ornithine | 12389 | 12389 |
| Leucine/Phenylalanine | 10449 | 10449 |
| Leucine/Phosphatidylcholine | 37936 | 3490112 |
| Leucine/Proline | 7614 | 7614 |
| Leucine/Serine | 19786 | 19786 |
| Leucine/Sphingomyelin | 27194 | 407910 |
| Leucine/Threonine | 7201 | 7201 |
| Leucine/Tryptophan | 12209 | 12209 |
| Leucine/Tyrosine | 9862 | 9862 |
| Leucine/Valine | 9862 | 9862 |
| Methionine/Ornithine | 18044 | 18044 |
| Methionine/Phenylalanine | 16695 | 16695 |
| Methionine/Phosphatidylcholine | 41910 | 3855720 |
| Methionine/Proline | 14314 | 14314 |
| Methionine/Serine | 23487 | 23487 |
| Methionine/Sphingomyelin | 33290 | 499350 |
| Methionine/Threonine | 13097 | 13097 |
| Methionine/Tryptophan | 16107 | 16107 |
| Methionine/Tyrosine | 17375 | 17375 |
| Methionine/Valine | 17375 | 17375 |
| Ornithine/Phenylalanine | 13651 | 13651 |
| Ornithine/Phosphatidylcholine | 42666 | 3925272 |
| Ornithine/Proline | 11728 | 11728 |
| Ornithine/Serine | 23324 | 23324 |
| Ornithine/Sphingomyelin | 32150 | 482250 |
| Ornithine/Threonine | 11974 | 11974 |
| Ornithine/Tryptophan | 16502 | 16502 |
| Ornithine/Tyrosine | 14655 | 14655 |
| Ornithine/Valine | 14655 | 14655 |
| Phenylalanine/Phosphatidylcholine | 41835 | 3848820 |
| Phenylalanine/Proline | 11052 | 11052 |
| Phenylalanine/Serine | 21438 | 21438 |
| Phenylalanine/Sphingomyelin | 31347 | 470205 |
| Phenylalanine/Threonine | 10338 | 10338 |
| Phenylalanine/Tryptophan | 13228 | 13228 |
| Phenylalanine/Tyrosine | 12489 | 12489 |
| Phenylalanine/Valine | 12489 | 12489 |
| Phosphatidylcholine/Proline | 38810 | 3570520 |
| Phosphatidylcholine/Serine | 42395 | 3900340 |
| Phosphatidylcholine/Sphingomyelin | 34731 | 47928780 |
| Phosphatidylcholine/Threonine | 35955 | 3307860 |
| Phosphatidylcholine/Tryptophan | 41184 | 3788928 |
| Phosphatidylcholine/Tyrosine | 41940 | 3858480 |
| Phosphatidylcholine/Valine | 41940 | 3858480 |
| Proline/Serine | 20006 | 20006 |
| Proline/Sphingomyelin | 28006 | 420090 |
| Proline/Threonine | 7155 | 7155 |
| Proline/Tryptophan | 13016 | 13016 |
| Proline/Tyrosine | 11811 | 11811 |
| Proline/Valine | 11811 | 11811 |
| Serine/Sphingomyelin | 31738 | 476070 |
| Serine/Threonine | 16657 | 16657 |
| Serine/Tryptophan | 22076 | 22076 |
| Serine/Tyrosine | 22311 | 22311 |
| Serine/Valine | 22311 | 22311 |
| Sphingomyelin/Threonine | 25277 | 379155 |
| Sphingomyelin/Tryptophan | 32681 | 490215 |
| Sphingomyelin/Tyrosine | 31476 | 472140 |
| Sphingomyelin/Valine | 31476 | 472140 |
| Threonine/Tryptophan | 11676 | 11676 |
| Threonine/Tyrosine | 11305 | 11305 |
| Threonine/Valine | 11305 | 11305 |
| Tryptophan/Tyrosine | 15561 | 15561 |
| Tryptophan/Valine | 15561 | 15561 |
| Tyrosine/Valine | 9633 | 9633 |
| Carnitine/Carnitine | 11239 | 9215980 |
| Phosphatidylcholine/Phosphatidylcholine | 31676 | 132595736 |
| Sphingomyelin/Sphingomyelin | 21290 | 2235450 |
| **Total** | **2969397** | **423645558** |

^1^ is the union of the SNP set generated for the metabolites in the numerator and denominator of the corresponding ratio. ^2^ In the case of aggregated compounds, the SNP set is multiplied by the number of compounds present in that class.

**Additional file 1: S4. Compounds filtered for the Kegg:Reaction Scheme**

cpd:C00001 H2O; Water

cpd:C00002 ATP; Adenosine 5'-triphosphate

cpd:C00003 NAD+; NAD; Nicotinamide adenine dinucleotide; DPN; Diphosphopyridine nucleotide; Nadide

cpd:C00004 NADH; DPNH; Reduced nicotinamide adenine dinucleotide

cpd:C00005 NADPH; TPNH; Reduced nicotinamide adenine dinucleotide phosphate

cpd:C00006 NADP+; NADP; Nicotinamide adenine dinucleotide phosphate; beta-Nicotinamide adenine dinucleotide phosphate; TPN; Triphosphopyridine nucleotide

cpd:C00007 Oxygen; O2

cpd:C00008 ADP; Adenosine 5'-diphosphate

cpd:C00009 Orthophosphate; Phosphate; Phosphoric acid; Orthophosphoric acid

cpd:C00010 CoA; Coenzyme A; CoA-SH

cpd:C00011 CO2; Carbon dioxide

cpd:C00012 Peptide

cpd:C00013 Diphosphate; Diphosphoric acid; Pyrophosphate; Pyrophosphoric acid; PPi

cpd:C00014 NH3; Ammonia

cpd:C00015 UDP; Uridine 5'-diphosphate

cpd:C00016 FAD; Flavin adenine dinucleotide

cpd:C00019 S-Adenosyl-L-methionine; S-Adenosylmethionine; AdoMet; SAM

cpd:C00020 AMP; Adenosine 5'-monophosphate; Adenylic acid; Adenylate; 5'-AMP; 5'-Adenylic acid; 5'-Adenosine monophosphate; Adenosine 5'-phosphate

cpd:C00024 Acetyl-CoA; Acetyl coenzyme A

cpd:C00027 Hydrogen peroxide; H2O2; Oxydol

cpd:C00028 Acceptor; Hydrogen-acceptor; A; Oxidized donor

cpd:C00030 Reduced acceptor; AH2; Hydrogen-donor; Donor

cpd:C00033 Acetate; Acetic acid; Ethanoic acid

cpd:C00035 GDP; Guanosine 5'-diphosphate; Guanosine diphosphate

cpd:C00040 Acyl-CoA; Acyl coenzyme A

cpd:C00044 GTP; Guanosine 5'-triphosphate

cpd:C00046 RNA; RNAn; RNAn+1; RNA(linear); (Ribonucleotide)n; (Ribonucleotide)m; (Ribonucleotide)n+m; Ribonucleic acid

cpd:C00055 CMP; Cytidine-5'-monophosphate; Cytidylic acid

cpd:C00063 CTP; Cytidine 5'-triphosphate; Cytidine triphosphate

cpd:C00067 Formaldehyde; Methanal; Oxomethane; Oxomethylene; Methylene oxide; Formalin

cpd:C00075 UTP; Uridine 5'-triphosphate; Uridine triphosphate

cpd:C00080 H+; Hydron

cpd:C00084 Acetaldehyde; Ethanal

cpd:C00086 Urea; Carbamide

cpd:C00091 Succinyl-CoA; Succinyl coenzyme A

cpd:C00105 UMP; Uridylic acid; Uridine monophosphate; Uridine 5'-monophosphate; 5'Uridylic acid

cpd:C00106 Uracil

cpd:C00112 CDP; Cytidine 5'-diphosphate; Cytidine diphosphate

cpd:C00125 Ferricytochrome c; Cytochrome c3+

cpd:C00126 Ferrocytochrome c; Cytochrome c2+; Reduced cytochrome c

cpd:C00131 dATP; 2'-Deoxyadenosine 5'-triphosphate; Deoxyadenosine 5'-triphosphate; Deoxyadenosine triphosphate

cpd:C00144 GMP; Guanosine 5'-phosphate; Guanosine monophosphate; Guanosine 5'-monophosphate; Guanylic acid

cpd:C00147 Adenine; 6-Aminopurine

cpd:C00151 L-Amino acid; L-2-Amino acid

cpd:C00161 2-Oxo acid; 2-Oxocarboxylate

cpd:C00162 Fatty acid

cpd:C00177 Cyanide; Prussiate; CN-; Cyano

cpd:C00212 Adenosine

cpd:C00178 Thymine; 5-Methyluracil

cpd:C00206 dADP; 2'-Deoxyadenosine 5'-diphosphate

cpd:C00214 Thymidine; Deoxythymidine

cpd:C00239 dCMP; Deoxycytidylic acid; Deoxycytidine monophosphate; Deoxycytidylate; 2'-Deoxycytidine 5'-monophosphate

cpd:C00240 rRNA; Ribosomal RNA

cpd:C00227 Acetyl phosphate

cpd:C00242 Guanine; 2-Amino-6-hydroxypurine

cpd:C00286 dGTP; 2'-Deoxyguanosine 5'-triphosphate; Deoxyguanosine 5'-triphosphate; Deoxyguanosine triphosphate

cpd:C00288 HCO3-; Bicarbonate; Hydrogencarbonate; Acid carbonate

cpd:C00299 Uridine

cpd:C00330 Deoxyguanosine; 2'-Deoxyguanosine

cpd:C00360 dAMP; 2'-Deoxyadenosine 5'-phosphate; 2'-Deoxyadenosine 5'-monophosphate; Deoxyadenylic acid; Deoxyadenosine monophosphate

cpd:C00361 dGDP; 2'-Deoxyguanosine 5'-diphosphate

cpd:C00362 dGMP; 2'-Deoxyguanosine 5'-monophosphate; 2'-Deoxyguanosine 5'-phosphate; Deoxyguanylic acid; Deoxyguanosine monophosphate

cpd:C00363 dTDP; Deoxythymidine 5'-diphosphate

cpd:C00364 dTMP; Thymidine 5'-phosphate; Deoxythymidine 5'-phosphate; Thymidylic acid; 5'-Thymidylic acid; Thymidine monophosphate; Deoxythymidylic acid; Thymidylate

cpd:C00365 dUMP; Deoxyuridylic acid; Deoxyuridine monophosphate; Deoxyuridine 5'-phosphate; 2'-Deoxyuridine 5'-phosphate

cpd:C00380 Cytosine

cpd:C00387 Guanosine

cpd:C00458 dCTP; Deoxycytidine 5'-triphosphate; Deoxycytidine triphosphate; 2'-Deoxycytidine 5'-triphosphate

cpd:C00459 dTTP; Deoxythymidine triphosphate; Deoxythymidine 5'-triphosphate; TTP

cpd:C00460 dUTP; 2'-Deoxyuridine 5'-triphosphate

cpd:C00475 Cytidine

cpd:C00512 S-Benzoate coenzyme A; Benzoyl-CoA

cpd:C00526 Deoxyuridine; 2-Deoxyuridine; 2'-Deoxyuridine

cpd:C00533 Nitric oxide; NO; Nitrogen monoxide

cpd:C00559 Deoxyadenosine; 2'-Deoxyadenosine

cpd:C00575 3',5'-Cyclic AMP; Cyclic adenylic acid; Cyclic AMP; Adenosine 3',5'-phosphate; Adenosine 3',5'-cyclic phosphate; cAMP

cpd:C00698 Cl-; Chloride; Chloride ion

cpd:C00705 dCDP; 2'-Deoxycytidine diphosphate; 2'-Deoxycytidine 5'-diphosphate

cpd:C00821 DNA adenine

cpd:C00856 DNA cytosine; Cytosine (in DNA)

cpd:C00881 Deoxycytidine; 2'-Deoxycytidine

cpd:C00941 3',5'-Cyclic CMP; Cytidine 3',5'-cyclic monophosphate

cpd:C00942 3',5'-Cyclic GMP; Guanosine 3',5'-cyclic monophosphate; Guanosine 3',5'-cyclic phosphate; Cyclic GMP; cGMP

cpd:C00943 3',5'-Cyclic IMP; Inosine 3',5'-cyclic monophosphate

cpd:C00968 3',5'-Cyclic dAMP

cpd:C01021 Aromatic amino acid; Aromatic L-amino acid

cpd:C01346 dUDP; 2'-Deoxyuridine 5'-diphosphate

cpd:C01352 FADH2

cpd:C01642 tRNA(Gly)

cpd:C01647 tRNA(Met)

cpd:C01764 tRNA containing uridine at position 54; tRNA UpsiC

cpd:C01794 Choloyl-CoA; 3alpha,7alpha,12alpha-Trihydroxy-5beta-cholanoyl-CoA; 3alpha,7alpha,12alpha-Trihydroxy-5beta-cholan-24-one-CoA

cpd:C01977 tRNA guanine

cpd:C02353 2',3'-Cyclic AMP

cpd:C02354 2',3'-Cyclic CMP

cpd:C02355 2',3'-Cyclic UMP

cpd:C02412 Glycyl-tRNA(Gly)

cpd:C02430 L-Methionyl-tRNA; L-Methionyl-tRNA(Met)

cpd:C02507 3',5'-Cyclic dGMP

cpd:C03110 DNA N4-methylcytosine

cpd:C03391 DNA 6-methylaminopurine

cpd:C03446 tRNA containing ribothymidine at position 54; tRNA TpsiC

cpd:C03395 Fatty acid methyl ester

cpd:C04152 rRNA containing N1-methylguanine

cpd:C04153 rRNA containing N2-methylguanine

cpd:C04154 rRNA containing N6-methyladenine; rRNA(N6-methyladenine)

cpd:C04156 tRNA containing N1-methyladenine

cpd:C04157 tRNA containing N1-methylguanine

cpd:C04158 tRNA containing N2-methylguanine

cpd:C04159 tRNA containing N6-methyladenine

cpd:C04160 tRNA containing N7-methylguanine

cpd:C04268 dTDP-4-amino-4,6-dideoxy-D-glucose

cpd:C04545 tRNA containing 2'-O-methylguanosine

cpd:C04728 tRNA containing 5-methylaminomethyl-2-thiouridylate; tRNA containing mnm5s2U

cpd:C04779 rRNA containing a single residue of 2'-O-methyladenosine

cpd:C05167 alpha-Amino acid

cpd:C05198 5'-Deoxyadenosine

cpd:C05337 Chenodeoxycholoyl-CoA; 3alpha,7alpha-Dihydroxy-5beta-cholanoyl-CoA

cpd:C05338 4-Hydroxyphenylacetyl-CoA

cpd:C05777 Coenzyme F430; Factor F430

cpd:C05359 e-; Electron

cpd:C06194 2',3'-Cyclic GMP

cpd:C11378 Ubiquinone-10; Ubidecarenone; Coenzyme Q10

cpd:C15670 Heme A

cpd:C15672 Heme O

cpd:C15817 Heme C

cpd:C11478 tRNA containing 5-aminomethyl-2-thiouridine; tRNA containing nm5s2U

cpd:C17023 Sulfur donor; S-donor

cpd:C17322 tRNA containing 2-thiouridine; tRNA containing s2U

cpd:C17323 tRNA containing 5-carboxymethylaminomethyl-2-thiouridine; tRNA containing cnm5s2U

cpd:C17324 tRNA adenine

cpd:C19637 Coenzyme M; 2,2'-Dithiodiethanesulfonic acid

**Additional file 1: Table S5 Metabolite specific break-up of the performance of database:interrogation schemes**

| **Arginine** | Size of Gene Set | Sensitivity | Genes with SNPs < 1E-02 | Number of Genes with SNPs < 1E-02 | Percentage of genes at 1E-02 |
| --- | --- | --- | --- | --- | --- |
| Biocyc_Pathway | 20 |  |  | 0 | 0 |
| Biocyc_Reaction | 104 |  | SLC32A1 | 1 | 0.96 |
| Kegg_Pathway | 57 |  |  | 0 | 0 |
| Kegg_Reaction | 179 |  |  | 0 | 0 |
|  |  |  |  |  |  |
| **Carnitine** | Size of Gene Set | Sensitivity | Genes with SNPs < 1E-02 | Number of Genes with SNPs < 1E-02 | Percentage of genes at 1E-02 |
| Biocyc_Pathway | 32 | 0.3 | ACADL,ACADM,ACSL1 | 3 | 9.38 |
| Biocyc_Reaction | 206 | 0.4 | ABHD6,ACADM,ACADS,ACSL1,AGPAT4,CRAT,DHTKD1,FADS1,FADS2,GPAM,HMGCS2,IDH3B,MCCC1,NMT2,P4HA2,PLA2G2A,PLA2G2E,SCD,SLC27A6,XYLT1 | 20 | 9.71 |
| Kegg_Pathway | 81 | 0.4 | ACADL,ACADM,ACADS,ACSL1,ADH1A,ADH1B,ADH1C,ADH7 | 8 | 9.88 |
| Kegg_Reaction | 94 | 0.3 | ACADL,ACADM,ACSL1,BAAT,CRAT,DHTKD1,IDH3B,P4HA2,PECR,PHGDH | 10 | 10.64 |
|  |  |  |  |  |  |
| **Glycine** | Size of Gene Set | Sensitivity | Genes with SNPs < 1E-02 | Number of Genes with SNPs < 1E-02 | Percentage of genes at 1E-02 |
| Biocyc_Pathway | 90 | 0.0 |  | 0 | 0 |
| Biocyc_Reaction | 192 | 0.0 | ALDH1L1 | 1 | 0.52 |
| Kegg_Pathway | 173 | 1.0 | CPS1 | 1 | 0.57 |
| Kegg_Reaction | 432 | 0.0 | ALDH1L1 | 1 | 0.23 |
|  |  |  |  |  |  |
| **Ornithine** | Size of Gene Set | Sensitivity | Genes with SNPs < 1E-02 | Number of Genes with SNPs < 1E-02 | Percentage of genes at 1E-02 |
| Biocyc_Pathway | 16 |  |  | 0 | 0 |
| Biocyc_Reaction | 150 |  |  | 0 | 0 |
| Kegg_Pathway | 103 |  |  | 0 | 0 |
| Kegg_Reaction | 159 | 1.0 | PHGDH | 1 | 0.63 |
|  |  |  |  |  |  |
| **Phosphatidylcholine** | Size of Gene Set | Sensitivity | Genes with SNPs < 1E-02 | Number of Genes with SNPs < 1E-02 | Percentage of genes at 1E-02 |
| Biocyc_Pathway | 188 | 0.4 | ACSL1,AGPAT1,FADS1,FADS2,GPAM,LRAT,MOGAT1,PLA2G4E,PLCB1,PLD1,PLD2,PNLIP,PPAPDC1A,PTGIS,RBP4,RLBP1,SCD | 17 | 9.04 |
| Biocyc_Reaction | 361 | 0.4 | ACSL1,ADH7,ADPRM,AGPAT1,ATP8A1,ATP8A2,ATP8B4,CBS,DGKQ,FADS1,FADS2,GPAM,LRAT,MBOAT1,MOGAT1,PLA2G4E,PLA2G7,PLCB1,PLD1,PLD2,PNLIP,PNPLA6,PPAPDC1A,PPT2,PTDSS1,PTGIS,RBP4,RLBP1,SCD,SLC27A6,SPTLC3,XYLT1 | 32 | 8.86 |
| Kegg_Pathway | 312 | 0.6 | ACADM,ACOT1,ACSL1,ADCY8,ADCY9,ADH7,ADPRM,AGPAT1,BAAT,CACNA1C,CNR1,DAGLA,DGKQ,ELOVL2,FADS1,FADS2,GABRB1,GABRB2,GABRR3,GNB4,GNGT1,GPAM,HSD17B12,KCNJ3,KCNJ6,MBOAT1,PECR,PLA2G4E,PLCB1,PLD1,PLD2,PNPLA6,PPT2,PRKCA,PRKCB,PTDSS1,PTGIS,SCD,SLC32A1,TECR | 40 | 12.82 |
| Kegg_Reaction | 343 | 0.2 | AADAC,ACADM,ACOT1,ACSL1,ADH7,ADPRM,AGPAT1,AHCYL2,BAAT,CBS,CERS4,DGKQ,ENPP2,GPAM,LRAT,MBOAT1,MLL,MOGAT1,PECR,PLA2G4E,PLCB1,PLD1,PLD2,PNLIP,PNLIPRP1,PNPLA6,PPT2,PTGIS,SCD,SOAT1,SOAT2,SPTLC3 | 32 | 9.33 |
|  |  |  |  |  |  |
| **Serine** | Size of Gene Set | Sensitivity | Genes with SNPs < 1E-02 | Number of Genes with SNPs < 1E-02 | Percentage of genes at 1E-02 |
| Biocyc_Pathway | 37 | 1.0 | PHGDH | 1 | 2.7 |
| Biocyc_Reaction | 135 |  |  | 0 | 0 |
| Kegg_Pathway | 152 | 1.0 | PHGDH,PSPH | 2 | 1.32 |
| Kegg_Reaction | 219 |  | ACOT1,PECR,PSPH | 3 | 1.37 |
|  |  |  |  |  |  |
| **Sphingomyelin** | Size of Gene Set | Sensitivity | Genes with SNPs < 1E-02 | Number of Genes with SNPs < 1E-02 | Percentage of genes at 1E-02 |
| Biocyc_Pathway | 160 |  | ACSL1,FADS1,FADS2,LRAT,PLCB1,PLD1,PLD2,PNLIP,PTGIS,SCD | 10 | 6.25 |
| Biocyc_Reaction | 331 | 1.0 | ACSL1,ATP8A1,DGKQ,FADS1,FADS2,LRAT,PLCB1,PLD1,PLD2,PNLIP,PPAPDC1A,PTGIS,SCD,SPTLC3 | 14 | 4.23 |
| Kegg_Pathway | 189 | 1.0 | ACSL1,CERS4,ELOVL2,FADS1,FADS2,HSD17B12,PTGIS,SCD,SPTLC3,TECR | 10 | 5.29 |
| Kegg_Reaction | 241 | 1.0 | ACSL1,CERS4,DGKQ,ENPP2,LRAT,PLCB1,PLD1,PLD2,PNLIP,PNLIPRP1,PTGIS,SCD,SOAT1,SPTLC3 | 14 | 5.81 |

Tables provide a metabolite specific result of our method for each of the four schemes shown in the first column. Sensitivity is a measure of the actual positives that have been captured by our method. Sensitivity is the ratio of number of top hits that our method could capture over the total number of top hits for that metabolite in the Illig *et al* study.

**Additional file 1: Table S6. Best case associations of loci with phosphatidylcholines in the Illig *et al* and Demirkan *et al* datasets**

| **Gene Symbol** | **SNP from the Illig et al dataset** | **Trait** | **p-value** | **SNP from the Demirkan et al dataset** | **Trait** | **p-value** |
| --- | --- | --- | --- | --- | --- | --- |
| *AADAC* | rs12152407 | PC aa C32:1 | 3.61E-04 | rs1520216 | PC aa 30:0 | 1.95E-04 |
| *ACOT1* | rs1014087 | lysoPC a C6:0 | 2.62E-04 | rs1014087 | PC ae 40:6 | 2.78E-03 |
| *ACSL1* | rs2046813 | PC ae C44:5 | 3.83E-04 | rs13142179 | PC aa 40:2 | 7.32E-05 |
| *ADCY8* | rs11786743 | PC ae C40:6 | 4.03E-05 | rs1560984 | PC aa 40:6 | 1.01E-05 |
| *ADCY9* | rs17136692 | PC aa C32:2 | 7.63E-04 | rs2601788 | PC ae 34:0 | 2.59E-05 |
| *ADH7* | rs1121886 | lysoPC a C17:0 | 5.18E-03 | rs7696921 | PC ae 40:5 | 2.53E-05 |
| *ADPRM* | rs201607 | PC ae C36:4 | 7.67E-03 | rs2662970 | PC aa 42:5 | 1.86E-04 |
| *AGPAT1* | rs3131294 | PC aa C38:6 | 2.50E-04 | rs1061808 | PC aa 32:0 | 7.78E-10 |
| *AHCYL2* | rs1061280 | PC aa C26:0 | 9.13E-04 | rs6971551 | PC aa 34:2 | 4.99E-05 |
| *ATP8A1* | rs1386617 | PC ae C34:3 | 2.80E-03 | rs7654745 | PC ae 38:5 | 2.59E-05 |
| *ATP8A2* | rs9511983 | PC aa C34:1 | 3.06E-03 | rs1409268 | PC aa 38:6 | 1.21E-05 |
| *ATP8B4* | rs6493397 | PC aa C32:1 | 1.11E-03 | rs11070742 | PC ae 32:0 | 7.07E-06 |
| *BAAT* | rs1572983 | PC aa C42:0 | 7.21E-04 | rs12342675 | PC aa 36:0 | 4.27E-04 |
| *CACNA1C* | rs733782 | PC ae C30:0 | 3.89E-05 | rs2239073 | LPC aa 18:0 | 1.65E-05 |
| *CBS* | rs719038 | PC ae C44:3 | 2.04E-06 | rs11700857 | PC aa 34:3 | 7.31E-05 |
| *CERS4* | rs4804286 | lysoPC a C6:0 | 7.22E-03 | rs12974685 | PC ae 32:1 | 6.43E-04 |
| *CNR1* | rs10485168 | PC ae C38:1 | 3.89E-05 | rs7738812 | PC aa 38:3 | 7.59E-05 |
| *DGKQ* | rs11723135 | PC ae C38:0 | 2.13E-03 | rs2290405 | PC aa 38:4 | 7.07E-04 |
| *ENPP2* | rs987534 | lysoPC a C20:3 | 2.28E-04 | rs2289885 | PC aa 36:5 | 5.91E-04 |
| *GABRB1* | rs7435958 | PC ae C34:0 | 1.26E-03 | rs3775534 | PC ae 36:0 | 2.07E-04 |
| *GABRB2* | rs2962394 | PC ae C30:1 | 3.06E-03 | rs17059514 | PC aa 36:5 | 9.54E-05 |
| *GABRR3* | rs13091233 | PC ae C40:5 | 2.12E-04 | rs13084407 | PC aa 38:1 | 2.15E-04 |
| *GNB4* | rs7622921 | PC ae C36:5 | 4.26E-03 | rs11715227 | PC ae 40:4 | 7.78E-04 |
| *GNGT1* | rs2157815 | PC aa C42:0 | 9.44E-05 | rs10230334 | PC ae 36:1 | 8.49E-04 |
| *GPAM* | rs2246253 | PC ae C34:3 | 1.25E-04 | rs2297991 | PC aa 34:3 | 5.73E-06 |
| *HSD17B12* | rs2862999 | PC aa C34:4 | 2.66E-05 | rs11037589 | PC aa 42:4 | 3.49E-05 |
| *KCNJ3* | rs12617694 | PC aa C30:2 | 4.73E-03 | rs17622700 | PC aa 32:0 | 7.43E-04 |
| *KCNJ6* | rs970641 | PC aa C42:5 | 3.68E-03 | rs2898334 | pc_mono | 1.21E-05 |
| *LRAT* | rs1541732 | PC ae C38:1 | 1.17E-03 | rs201823 | pc_mono | 1.48E-04 |
| *MBOAT1* | rs9465673 | PC aa C36:6 | 9.64E-05 | rs9348414 | PC ae 40:5 | 4.36E-05 |
| *MLL* | rs548877 | PC aa C36:5 | 3.92E-03 | rs1079118 | LPC 22:4 | 5.64E-04 |
| *MOGAT1* | rs4234043 | PC ae C34:0 | 2.50E-04 | rs7575668 | LPC 22:6 | 1.23E-04 |
| *PECR* | rs3770536 | PC ae C40:6 | 2.02E-05 | rs3770558 | PC aa 40:4 | 4.48E-05 |
| *PLA2G4E* | rs1530837 | PC aa C32:1 | 7.60E-04 | rs16972296 | PC ae 42:5 | 7.77E-05 |
| *PLA2G7* | rs9395212 | lysoPC a C20:4 | 3.86E-03 | rs7760667 | PC aa 34:2 | 3.57E-04 |
| *PLCB1* | rs6056188 | PC aa C30:0 | 9.55E-06 | rs2223538 | PC aa 34:3 | 2.90E-05 |
| *PLD1* | rs13065846 | PC aa C42:5 | 8.08E-04 | rs4894769 | PC ae 38:4 | 7.47E-05 |
| *PLD2* | rs9890937 | PC aa C38:1 | 3.62E-03 | rs3764897 | PC aa 42:5 | 7.15E-05 |
| *PNLIP* | rs3010494 | PC ae C34:1 | 3.87E-03 | rs4523611 | PC aa 36:1 | 7.39E-06 |
| *PNLIPRP1* | rs3010494 | PC ae C34:1 | 3.87E-03 | rs10885997 | PC aa 36:1 | 7.88E-09 |
| *PNPLA6* | rs4804750 | PC aa C40:2 | 7.96E-05 | rs4804750 | LPC 20:4 | 4.56E-05 |
| *PPAPDC1A* | rs2463148 | PC aa C38:5 | 4.01E-04 | rs10749377 | PC aa 40:2 | 8.55E-05 |
| *PPT2* | rs3131294 | PC aa C38:6 | 2.50E-04 | rs1269852 | PC aa 32:0 | 3.27E-04 |
| *PRKCA* | rs4254365 | lysoPC a C28:1 | 1.03E-03 | rs12946615 | PC aa 38:5 | 1.36E-05 |
| *PRKCB* | rs16940401 | PC ae C34:0 | 6.05E-03 | rs2238498 | PC aa 38:0 | 2.15E-04 |
| *PTDSS1* | rs7842166 | PC ae C38:0 | 1.20E-03 | rs4734338 | pc_sat | 1.74E-04 |
| *PTGIS* | rs5629 | lysoPC a C28:0 | 2.79E-03 | rs562954 | PC aa 30:0 | 2.56E-04 |
| *RBP4* | rs10882265 | lysoPC a C16:0 | 2.51E-03 | rs7070454 | PC aa 40:7 | 1.11E-04 |
| *RLBP1* | rs2283436 | PC aa C42:6 | 8.09E-04 | rs12914337 | PC aa 38:2 | 1.26E-03 |
| *SCD* | rs603424 | PC ae C40:4 | 1.99E-04 | rs603424 | LPC 16:1 | 1.91E-08 |
| *SLC27A6* | rs6595832 | PC ae C40:5 | 2.87E-05 | rs4836421 | LPC 18:0 | 4.20E-04 |
| *SLC32A1* | rs6123987 | lysoPC a C18:0 | 4.67E-03 | rs6071173 | PC aa 38:4 | 1.72E-05 |
| *SOAT1* | rs12140800 | PC ae C40:1 | 1.51E-03 | rs11807253 | LPC 20:4 | 1.28E-05 |
| *SOAT2* | rs7298527 | lysoPC a C16:1 | 1.09E-03 | rs9658618 | PC ae 42:5 | 2.51E-04 |
| *SPTLC3* | rs168622 | PC ae C40:2 | 4.46E-05 | rs608994 | PC ae 36:1 | 1.95E-05 |
| *TECR* | rs7252966 | PC aa C32:0 | 1.69E-05 | rs4926222 | PC ae 36:0 | 4.81E-04 |
| *XYLT1* | rs28709752 | PC aa C38:0 | 6.16E-03 | rs8046171 | PC ae 34:1 | 6.45E-05 |

**Additional file 1: Table S7. Pleiotropic effect for phosphatidylcholines at select loci**

| **Gene Symbol** | **SNP from the Illig *et al* dataset** | **Trait** | **p-value** | **SNP from the Demirkan et al dataset** | **Trait** | **p-value** | **R^2^ between SNPs** |
| --- | --- | --- | --- | --- | --- | --- | --- |
| *CBS* | rs2124458 | PC ae C44:3 | 3.08E-06 | rs2124458 | LPC 18:3 | 4.00E-03 | 1 |
| *ADCY8* | rs11786743 | PC ae C40:6 | 4.03E-05 | rs11782768 | PC aa 40:4 | 2.43E-03 | 1 |
| *PNPLA6* | rs4804750 | PC aa C40:2 | 7.96E-05 | rs11259999 | LPC 20:4 | 5.67E-05 | 0.98 |
| *HSD17B12* | rs2862999 | PC aa C34:4 | 2.66E-05 | rs7111355 | PC ae 36:1 | 5.76E-03 | 0.98 |
| *GABRB2* | rs2962394 | PC ae C30:1 | 3.06E-03 | rs2910303 | PC 30:0 | 2.29E-04 | 0.99 |
| *LRAT* | rs1541732 | PC ae C38:1 | 1.17E-03 | rs201824 | PC 34:1 | 1.54E-04 | 0.96 |
| *PLCB1* | rs909895 | PC ae C44:5 | 1.94E-04 | rs17446441 | PC O 38:4 | 3.99E-03 | 0.85 |
| *GPAM* | rs2246253 | PC ae C34:3 | 1.25E-04 | rs4918723 | PC 34:2 | 6.87E-03 | 0.78 |
| *SOAT1* | rs1044925 | PC ae C40:1 | 1.62E-03 | rs3753525 | PC O 40:5 | 2.11E-04 | 0.76 |
